# H3K4me3 Genome-Wide Distribution and Transcriptional Regulation of Transposable Elements by RNA Pol2 Deposition

**DOI:** 10.3390/ijms252413545

**Published:** 2024-12-18

**Authors:** Xiaowei Chen, Hua Yang, Liqin Wang, Ying Chen, Yingnan Yang, Haonan Chen, Feng Wang, Yanli Zhang, Mingtian Deng

**Affiliations:** 1 Jiangsu Livestock Embryo Engineering Laboratory, College of Animal Science and Technology, Nanjing Agricultural University, Nanjing 210095, China; 2022105010@stu.njau.edu.cn (X.C.); t2023004@njau.edu.cn (H.Y.); 2023205022@stu.njau.edu.cn (Y.Y.); chnan@stu.njau.edu.cn (H.C.); caeet@njau.edu.cn (F.W.); 2 Key Laboratory of Genetics Breeding and Reproduction of Grass Feeding Livestock, Ministry of Agriculture and Rural Affairs, Urumqi 830000, China; wlq6304@126.com (L.W.); cy08272024@163.com (Y.C.)

**Keywords:** H3K4me3, transposable elements, RNA Pol2, zygotic genome activation, embryonic stem cells

## Abstract

Zygotic genome activation (ZGA) is critical for early embryo development and is meticulously regulated by epigenetic modifications. H3K4me3 is a transcription-permissive histone mark preferentially found at promoters, but its distribution across genome features remains incompletely understood. In this study, we investigated the genome-wide enrichment of H3K4me3 during early embryo development and embryonic stem cells (ESCs) in both sheep and mice. We discovered that broad H3K4me3 domains were present in MII stage oocytes and were progressively diminished, while promoter H3K4me3 enrichment was increased and correlated with gene upregulation during ZGA in sheep. Additionally, we reported the dynamic distribution of H3K4me3 at the transposable elements (TEs) during early embryo development in both sheep and mice. Specifically, the H3K4me3 distribution of LINE1 and ERVL, two subsets of TEs, was associated with their expression during early embryo development in sheep. Furthermore, H3K4me3 enrichment in TEs was greatly increased during ZGA following Kdm5b knockdown, and the distribution of RNA polymerase II (Pol2) in TEs was also markedly increased in Kdm5b knockout ESCs in mice. These findings suggest that H3K4me3 plays important roles in regulating TE expression through interaction with RNA Pol2, providing valuable insights into the regulation of ZGA initiation and cell fate determination by H3K4me3.

## 1. Introduction

Mammalian pre-implantation development is a complex and critical process, including zygotic genome activation (ZGA) and cell fate determination. Increasing evidence has revealed that ZGA is essential for early embryo development in mammals [1,2]. During ZGA, developmental control transitions from maternally provided gene products to those newly synthesized from the zygotic genome [3,4]. The developmental control transition varies as the time of ZGA differs from species to species: 2-cell stage in mice, 4-/8-cell stage in humans and pigs, and 8-/16-cell stage in bovine, goats, and sheep [3,5,6]. The differences between sheep and other species are seldom explored.

Epigenetic modifications, such as H3K4me3, H2AZ, and H2A119ub, play a crucial role in the fine regulation of ZGA in mice [7,8,9,10]. H3K4me3 is a well-established transcription-permissive histone mark preferentially located at promoters. In mouse embryos, the genome-wide H3K4me3 signal coverage in oocytes rapidly decreases from the early 2-cell stage to the late 2-cell stage. Knockout of Kdm5b and Kdm5c, two H3K4me3 demethylases, leads to developmental arrest at the ZGA in mice [11]. In farm animals, although several studies and our previous data revealed dynamic changes in global H3K4me3 during early embryo development in bovine and goats by immunofluorescence staining [12,13,14], the genome-wide H3K4me3 distribution has been much less investigated due to the limited number of available embryos.

Embryonic stem cells (ESCs) derived from the inner cell mass (ICM) exhibit the unique properties of pluripotency and self-renewal [15,16]. Various research groups have generated induced blastoids using human, mouse, and bovine ESCs [17,18,19,20], suggesting that ESCs are an ideal model for studying cell fate determination. Previous studies have demonstrated that cell fate determination is largely an epigenetic process [21]. Kdm5b and H3K4me3 were reported to regulate mouse ESC differentiation and reprogramming [22,23,24]. However, the distribution status of H3K4me3 in sheep ESCs has not been determined.

Transposable elements (TEs), which constitute 25–50% of the entire genome in mammals [25], are broadly categorized into long terminal repeats (LTRs), long interspersed nuclear elements (LINEs), and short interspersed nuclear elements (SINEs) [26]. Recent studies have documented that TEs make contributions to genomic evolution, genome organization, and gene regulation [27,28]. During mouse ZGA, endogenous retrovirus-L (ERVL, one of the subsets of LTRs) is specifically activated, and depletion of ERVL transcripts leads to embryonic lethality due to defects in early lineage specification and genome stability [29]. LINE1 was activated on ZGA initiation and is integral to the mouse developmental program by the regulation of global chromatin accessibility [30]. TEs also play pivotal roles in cell fate determination in embryonic stem cells (ESCs). He et al. reported that ERVL and MT2_Mm are used to identify rare 2C-like subpopulations of cells in mice [31], and Zhang found that knockdown of LINE1 RNA induces the reversion of naive human ESCs to 8C-like cells [32]. Although TEs are repressed by different epigenetic mechanisms to prevent harmful insertion in the genome, the distribution status of H3K4me3 in TEs has not been investigated.

In the present study, we analyzed the genome-wide profiling of H3K4me3 during early embryo development and ESCs. We revealed broad H3K4me3 domain removal and promoter H3K4me3 increment and its correlation with gene upregulation during ZGA. We also reported the dynamic distribution of H3K4me3 at the TEs during early embryo development in both sheep and mice. Furthermore, H3K4me3 enrichment in TEs was greatly increased during ZGA following Kdm5b knockdown, and the distribution of RNA polymerase II (Pol2) in TEs was also markedly increased in Kdm5b knockout ESCs in mice.

## 2. Results

### 2.1. Genome-Wide Profiling of H3K4me3 During Sheep Early Embryo Development

**Figure 1:**
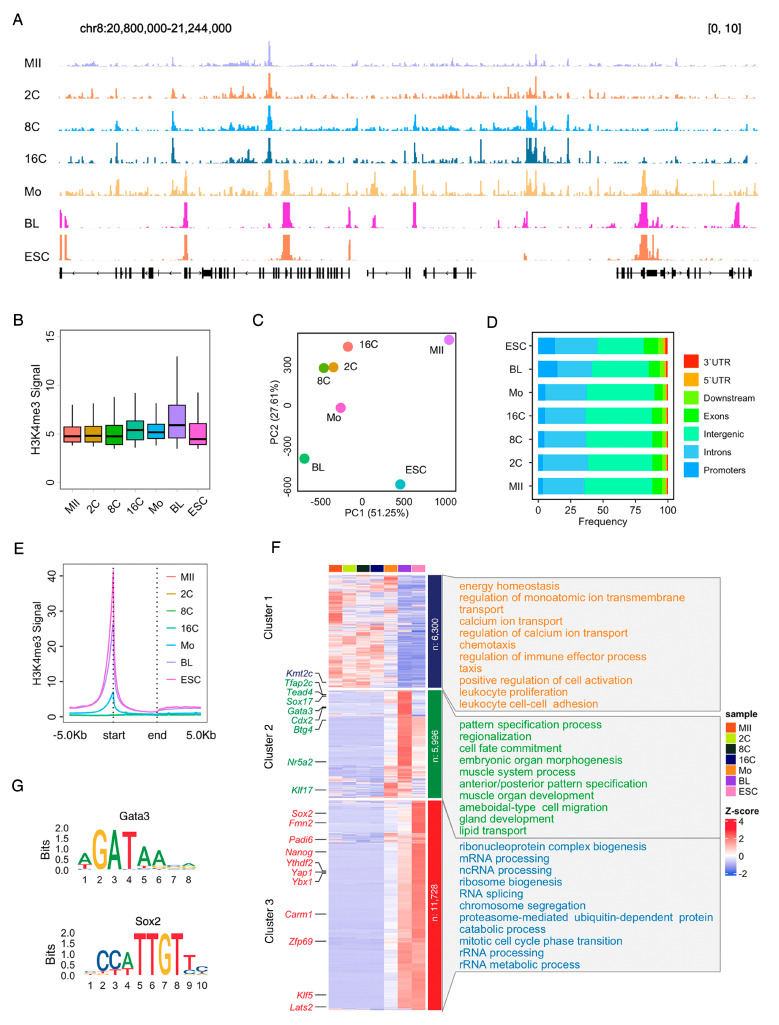
Genome-wide profiling of H3K4me3 during sheep early embryo development. (**A**) Snapshot of H3K4me3 distribution in chromosome 8 in the oocyte, 2-, 8-, and 16-cell stage embryos, morula embryos, blastocyst embryos, and ESCs in sheep. (**B**) Boxplot of H3K4me3 signals in sheep early embryos. (**C**) PCA plot showed that H3K4me3 enriched regions of 2-, 4-, 8-, and 16-cell stage embryos, MII stage oocytes, morula, blastocyst, and ESCs were clearly separated from each other. (**D**) Frequency of H3K4me3 enrichment of the early embryo in the genome features. (**E**) Metaplot of H3K4me3 in transcription start site (start) and transcription end sites (end) in the oocyte, 2-, 8-, and 16-cell stage embryos, morula embryos, blastocyst embryos, and ESCs in sheep. (**F**) Heatmap and Gene Ontology analysis of the promoter H3K4me3 in the oocyte, 2-, 8-, and 16-cell stage embryos, morula embryos, blastocyst embryos, and ESCs in sheep. (**G**) Motif analysis revealed H3K4me3 enrichment in Gata3 in blastocyst and Sox2 in ESC. MII, MII stage oocytes; 2C/8C/16C, 2-, 8-, and 16-cell stage embryos; Mo, morula embryos; BL, blastocyst embryos; ESC, embryonic stem cell.

We conducted a comprehensive analysis of H3K4me3 distribution during the early stages of sheep embryo development using CUT&Tag. As depicted in Figure 1A, H3K4me3 peaks were clearly observed across all embryonic stages and ESCs at the chromosome 8 regions, as revealed by the IGV browser. Boxplot revealed a significant increase in H3K4me3 signals from 8- to 16-cell stage embryos and from the morula to the blastocyst stage (Figure 1B). The PCA plot demonstrated that H3K4me3 enriched regions of 2-, 4-, 8-, and 16-cell stage embryos, MII stage oocytes, morula, blastocyst, and ESCs were clearly distinguished from each other, suggesting that genome-wide H3K4me3 modifications are dynamically changed and correlated with early embryo development in sheep (Figure 1C).

Given that H3K4me3 distribution in promoters is indicative of active gene expression, we next analyzed the genome-wide enrichment of H3K4me3 during early embryo development. H3K4me3 peaks were mapped to various genome features, including promoters, 5′ untranslated regions (UTRs), 3′ UTRs, exons, introns, intergenic introns, and immediate downstream regions. Over half of the H3K4me3 peaks were located in gene regions in all-stage embryos and ESCs (Figure 1D). In promoter regions, H3K4me3 enrichment was gradually increased from the MII stage oocytes to the blastocyst but decreased in the ESCs, as revealed by the metaplot (Figure 1E).

Using k-means algorithms, promoter H3K4me3 distribution in early embryos and ESCs were classified into three distinct clusters: oocyte-specific accumulation (cluster 1, n = 6300), including Kmt2c; blastocyst-specific accumulation (cluster 2, n = 5996), such as Tfap2c, Tead4, Cdx2, and Gata3, suggesting that these regions are related to trophectoderm (TE); ESC-specific accumulation (cluster 3, n = 11,728), including Sox2, Nanog, and Klf5, suggesting the regions are related to ICM. Gene Ontology (GO) analysis indicated that promoter H3K4me3 in cluster 1 was deposited for genes involved in calcium ion transport and positive regulation of cell activation. Promoter H3K4me3 in cluster 2 was deposited for genes enriched in pattern specification process regionalization, cell fate commitment, embryonic organ morphogenesis, and gland development. Promoter H3K4me3 in cluster 3 was deposited for genes involved in ribonucleoprotein complex biogenesis, RNA processing, ribosome biogenesis, RNA splicing, chromosome segregation, and RNA metabolism (Figure 1F). Motif analysis confirmed the H3K4me3 enrichment in Gata3 and Sox2 in blastocysts (Figure 1G). Collectively, these results suggest that promoter H3K4me3 accumulation is associated with gene expression for diverse biological functions during early embryo development.

### 2.2. Removal of H3K4me3 Broad Domains During Early Embryo Development

**Figure 2:**
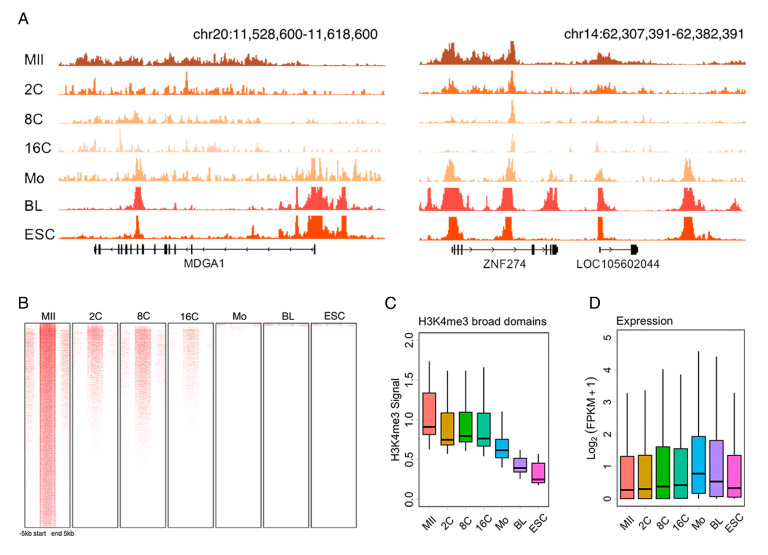
Removal of H3K4me3 broad domains during early embryo development. (**A**) Snapshot revealing that H3K4me3 broad domains occurred in sheep MII stage oocytes and were shifted to sharp peaks after fertilization. (**B**,**C**) Heatmaps and boxplot showing removal of H3K4me3 broad domains during early embryo development. (**D**) The distribution of H3K4me3 broad domain-deposited genes during early embryo development in sheep.

H3K4me3 broad domains are known to occur in mouse oocytes and to be severed as a critical epigenetic signature during early embryo development. A snapshot of MDGA1 and ZNG274 revealed that H3K4me3 broad domains also occurred in sheep MII stage oocytes and were shifted to sharp peaks post-fertilization. The H3K4me3 signal reached its nadir at the 16-cell stage and was then increased from the morula to the blastocyst stage (Figure 2A, Supplementary Table S1).

To identify broad H3K4me3 domains in sheep oocytes, we performed broad peak calling for H3K4me3 in oocytes based on MACS2, and regions greater than 5000 bp were defined as broad H3K4me3 domains. The broad H3K4me3 domains were gradually removed from the 2- to 16-cell stage embryos and were scarcely observed after ZGA (Figure 2B). In addition, the signal intensity of broad H3K4me3 domains was decreased from the MII stage oocytes to the blastocyst during early embryo development in sheep (Figure 2C). The boxplot revealed that the expression of genes marked by broad H3K4me3 domains was increased from MII stage oocytes to the 16-cell stage and sharply increased from the 16-cell stage embryos to the morula (Figure 2D).

### 2.3. Increased H3K4me3 Distribution Correlates with Gene Upregulation During ZGA

**Figure 3:**
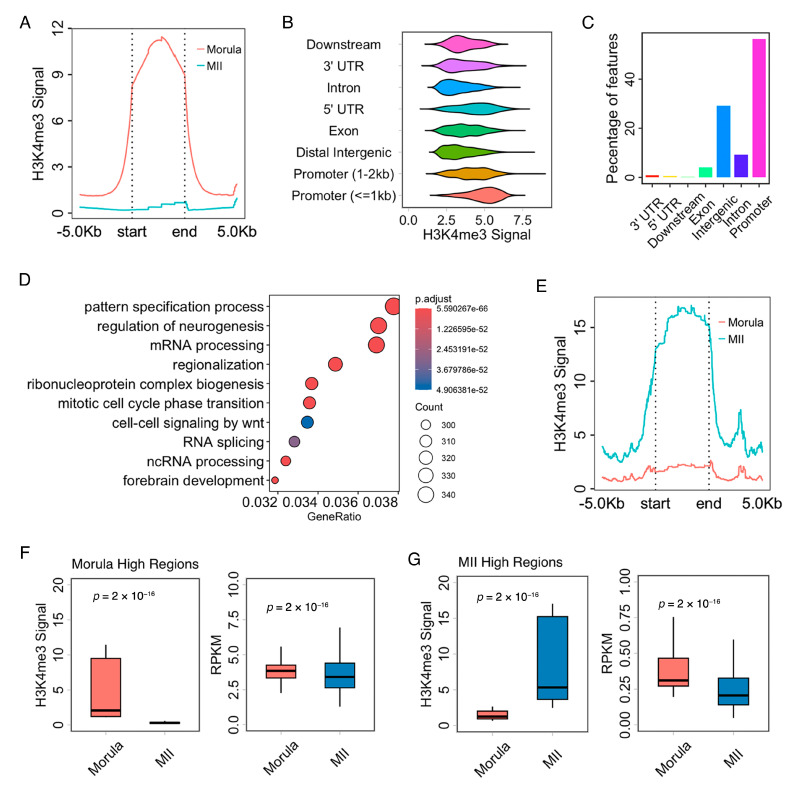
Increased H3K4me3 distribution correlates with gene upregulation during ZGA (**A**) Identification of morula-higher H3K4me3 peaks by comparing the distribution of H3K4me3 in the MII stage oocytes and the morula embryos. (**B**) Morula-specific H3K4me3 signal in various genome features. (**C**) Frequency of morula-higher H3K4me3 regions in the genome features. (**D**) Gene Ontology analysis of morula-higher H3K4me3 peaks. (**E**) Identification of oocyte-higher H3K4me3 peaks by comparing the distribution of H3K4me3 in the MII stage oocytes and the morula embryos. (**F**) Boxplot of H3K4me3 signal in morula-higher peaks and gene expression of morula-higher peak-deposited genes in the MII stage oocytes and the morula embryos. (**G**) Boxplot of H3K4me3 signal in oocyte-higher peaks and gene expression of morula-higher peak-deposited genes in the MII stage oocytes and the morula embryos.

ZGA occurred at the 16-cell stage embryos in sheep (in preparation). To explore the dynamic changes in H3K4me3 during ZGA in sheep, we compare the distribution of H3K4me3 between MII stage oocytes and morula embryos. The morula-specific H3K4me3 regions were enriched in promoters, 5′ UTRs, 3′ UTRs, exons, introns, intergenic introns, and immediate downstream regions (Figure 3A,B, Supplementary Table S2), and more than half of the H3K4me3 was deposited in the promoter (Figure 3C).

GO analysis revealed that the morula-specific H3K4me3 in the promoter was deposited for genes involved in the pattern-specification process, mRNA processing, ribonucleoprotein complex biogenesis, RNA splicing, and forebrain development (Figure 3D). In addition, many regions also lost the H3K4me3 distribution in the morula (Figure 3E). Specifically, genes with a higher H3K4me3 signal in the morula showed increased expression compared to the MII stage oocytes. Conversely, oocyte-specific H3K4me3 region-deposited genes showed a lower level of expression in the MII stage oocytes (Figure 3G), suggesting that the oocyte-specific H3K4me3 regions might represent broad domains that inhibit gene expression.

### 2.4. Dynamic H3K4me3 Changes at TEs During Early Embryo Development

**Figure 4:**
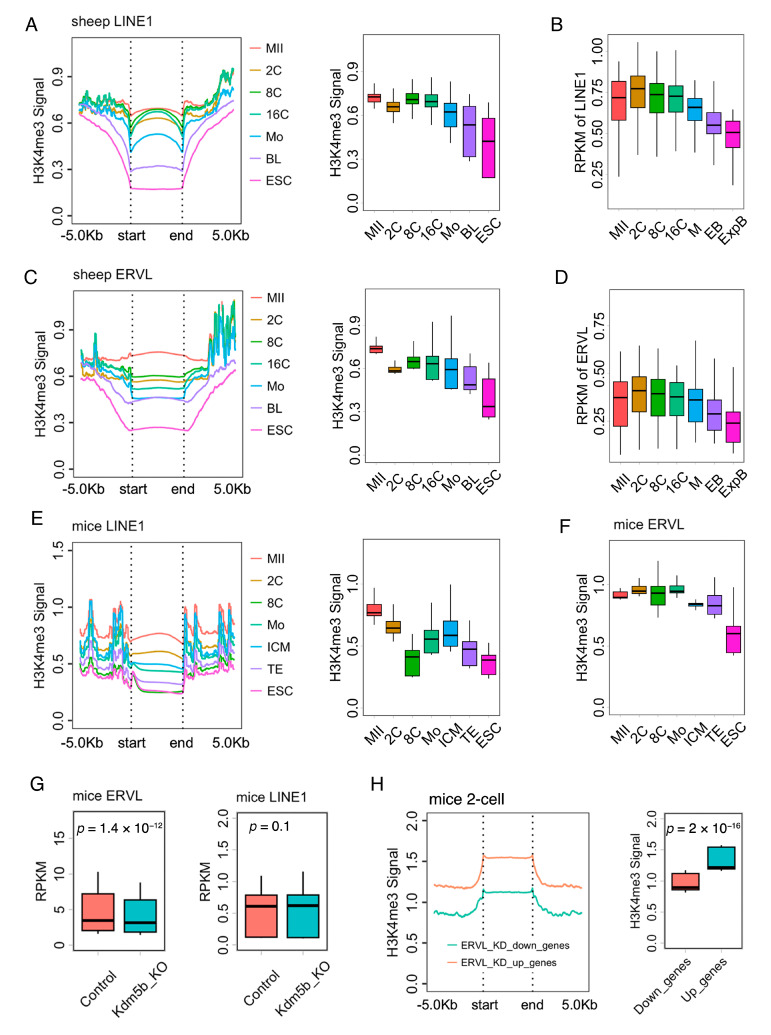
Dynamic H3K4me3 changes at TEs during early embryo development. (**A**) Metaplot and boxplot of H3K4me3 in LINE1 in oocyte, 2-, 8-, and 16-cell stage embryos, morula embryos, blastocyst embryos, and ESCs in sheep. (**B**) Expression of LINE1 during early embryo development in sheep. (**C**) Metaplot and boxplot of H3K4me3 in ERVL in oocytes, 2-, 8-, and 16-cell stage embryos, morula embryos, blastocyst embryos, and ESCs in sheep. (**D**) Expression of ERVL during early embryo development in sheep. (**E**) Metaplot and boxplot of H3K4me3 in LINE1 in oocytes, 2-, and 8-cell stage embryos, morula embryos, ICM. TE, and ESCs in mice. (**F**) Metaplot and boxplot of H3K4me3 in ERVL in oocytes, 2-, and 8-cell stage embryos, morula embryos, ICM, TE, and ESCs in mice. (**G**) Expression of LINE1 and ERVL in the control and the Kdm5b knockdown embryos at the 2-cell stage. (**H**) Metaplot and boxplot of H3K4me3 in upregulation and/or downregulation genes after ERVL was knocked down in mouse 2-cell stage embryos. MII, MII stage oocytes; 2C/8C/16C, 2-, 8-, and 16-cell stage embryos; Mo, morula embryos; BL, blastocyst embryos; ICM, inner cell mass; TE, trophectoderm; ESC, embryonic stem cell. RPKM, Reads Per Kilobase per Million mapped reads.

LINE1 and ERVL have been reported as critical factors during mouse pre-implantation development. We next analyzed H3K4me3 enrichment in LINE1 and ERVL during early embryo development in sheep. As shown in Figure 4A, the H3K4me3 signal in LINE1 was decreased from the MII stage oocytes to the 2-cell stage embryos, increased at the 8-cell stage embryos, and then sharply decreased from the 8-cell stage embryos to the blastocysts, showing the lowest level in the ESCs in sheep (Figure 4A). The expression of LINE1 was increased at the 2-cell stage embryos and maintained at the 8- and 16-cell stage embryos but decreased from the 16-cell stage embryos to the blastocysts (Figure 4B). As for ERVL, the H3K4me3 signal was sharply decreased after fertilization, further decreased during ZGA, and reached its lowest level in ESCs (Figure 4C). Moreover, the expression of ERVL was also decreased during ZGA (Figure 4D).

To confirm the conserved dynamic changes of H3K4me3 distribution in the TEs, we analyzed published H3K4me3 ChIP-seq data of mouse early embryo development. The H3K4me3 enrichment in LINE1 was decreased from the MII stage oocytes to the 8-cell stage embryos but was increased at the morula embryos and ICMs and decreased at the TEs and ESCs (Figure 4E). As for the ERVL, the H3K4me3 signal was maintained in the MII stage oocytes and the pre-implantation embryos but decreased in the ESCs (Figure 4F).

To study the relationship between the H3K4me3 distribution and the expression of TEs, we analyzed published RNA-seq data from mouse Kdm5b knockdown 2-cell stage embryos. ERVL was surprisingly down-regulated in mouse Kdm5b knockdown 2-cell stage embryos, while the expression of LINE1 showed no significant change after Kdm5b knockdown (Figure 4G). Since TEs play critical roles in gene expression regulation, we analyzed the H3K4me3 distribution in differential expression genes after ERVL knockdown. As expected, the up-regulated genes after ERVL knockdown showed more H3K4me3 enrichment than that of down-regulated genes in mouse 2-cell embryos (Figure 4H, Supplementary Table S3), suggesting that ERVL might crosstalk with H3K4me3 in fine-tuning the regulation of gene expression during early embryo development.

### 2.5. Increment RNA Pol2 Distribution in TEs in Kdm5b Knockout ESCs

**Figure 5:**
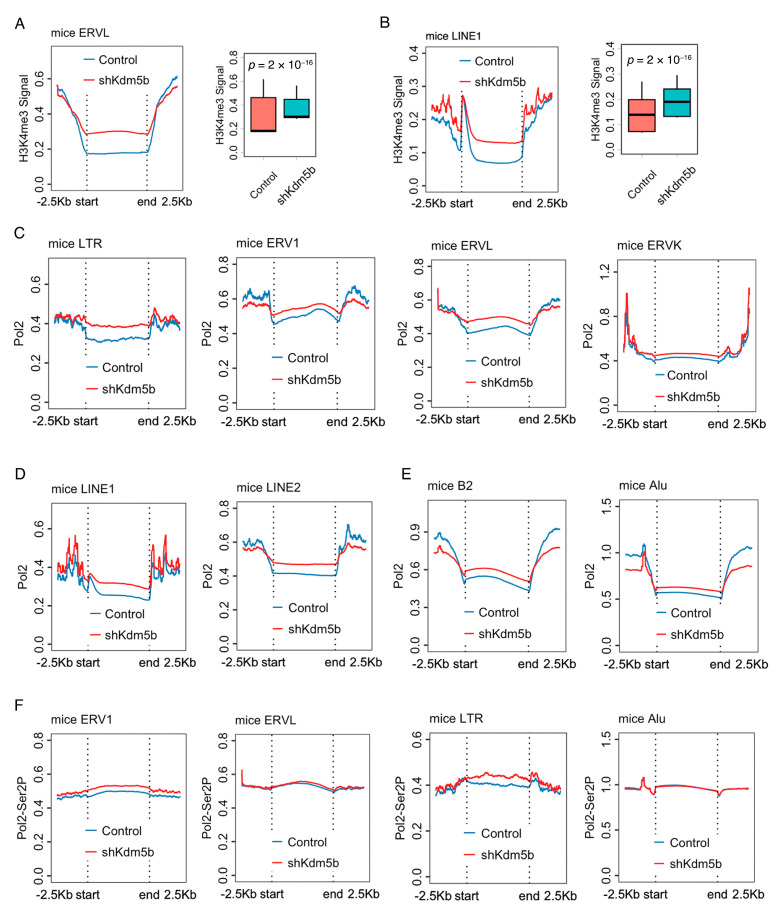
Increment RNA Pol2 distribution in TEs in Kdm5b knockout ESCs. (**A**,**B**) Metaplot and boxplot of H3K4me3 in LINE1 and/or ERVL in Kdm5b knockdown ESCs. (**C**) Metaplot and boxplot of RNA Pol2 in LTR, ERV1, ERVL, and ERVK in Kdm5b knockdown ESCs. (**D**) Metaplot and boxplot of RNA Pol2 in LINE1 and LINE2 in Kdm5b knockdown ESCs. (**E**) Metaplot and boxplot of RNA Pol2 in SINE B2 and Alu in Kdm5b knockdown ESCs. (**F**) Metaplot and boxplot of RNA Pol2 Ser2P in ERV1, ERVL, LTR, and Alu in Kdm5b knockdown ESCs.

H3K4me3 was reported to regulate RNA Pol2 promoter-proximal pause–release [33,34]. To reveal the sophisticated transcript regulation in TEs, we analyzed the distribution of RNA Pol2 in Kdm5b knockout ESCs. As expected, the Kdm5b knockdown resulted in an H3K4me3 increment in ERVL and LINE1 in mouse ESCs (Figure 5A,B). This was accompanied by a significant increase in RNA Pol2 distribution in LTR, ERV1, and ERVL in Kdm5b knockdown ESCs (Figure 5C). In LINEs, the knockdown of Kdm5b resulted in higher enrichment of RNA Pol2 in the LINE1 and LINE2 body (Figure 5D). In SINEs, RNA Pol2 distribution was also increased in the B2 and Alu of Kdm5b knockdown ESCs (Figure 5E). Of interest, RNA Pol2 showed less enrichment from 2.5 kb upstream of the TSS to the TSS and from the TES to the 2.5 kb downstream regions of ERV1, LINE2, B2, and Alu (Figure 5C–E).

RNA Pol2-Ser2P plays a role in the regulation of elongation during gene transcription. The distribution of RNA Pol2-Ser2P was also increased in ERV1 and LTR after Kdm5b knockdown in mouse ESCs, but there was no difference in ERVL and Alu (Figure 5F). Taken together, these findings suggest that H3K4me3 regulates RNA Pol2 distribution in TEs and is essential for the translational control of TEs.

## 3. Discussion

Due to the lack of a chromatin analysis approach in sparse samples, the investigation of the underlying mechanisms has been hindered. Using CUT&Tag, we analyzed the genome-wide distribution of H3K4me3 during early embryo development and ESCs in sheep. We revealed broad H3K4me3 domain removal and promoter H3K4me3 increment and its correlation with gene upregulation during ZGA. We also reported the dynamic distribution of H3K4me3 at the TEs during early embryo development in both sheep and mice. Furthermore, H3K4me3 enrichment in TEs was greatly increased during ZGA following Kdm5b knockdown, and the distribution of RNA polymerase II (Pol2) in TEs was also markedly increased in Kdm5b knockout ESCs in mice. Our data highlight the functional importance of H3K4me3 in RNA Pol2 distribution in TEs during early embryo development and in ESCs.

Broad H3K4me3 domains are a signature of constitutive expression of cell-type-specific regulation [35]. In mice, broad H3K4me3 domains were reported to protect the maternal genome from DNA methylation in oocytes and were rapidly removed after fertilization. During ZGA initiation, 86% of the zygotic genes overlapped with the broad H3K4me3 domains [11]. In bovine, Dang et al. reported the removal of broad H3K4me3 domains during early embryo development [14]. Consistently, we also found the removal of broad H3K4me3 domains after fertilization in sheep. These data suggested that gene expression regulation by broad H3K4me3 domains is a conserved mechanism during the maternal-to-zygotic transition progress in mammals.

Promoter H3K4me3 distribution is a classic transcription-permissive hallmark. During ZGA in mice and bovine, Liu et al. and Dang et al. reported increased enrichment of H3K4me3 at promoter regions during ZGA [11,14], which is consistent with our data. Given the massive gene expression during ZGA, the enhanced H3K4me3 in the promoter regions is highly likely to be a permit for transcription in sheep. Indeed, we found upregulation of genes with more enriched H3K4me3 in the promoter, supporting the notion that increased promoter H3K4me3 distribution is correlated with gene upregulation. Using immunofluorescence staining, the global H3K4me3 signal has been well documented during early embryo development in mice, bovine, pigs, and goats [12,14,36,37,38]. Contrary to the removal of global H3K4me3 during ZGA, we found increased promoter H3K4me3 distribution in sheep. This inconsistency has also been reported during ZGA in mice and bovine [11,14], suggesting that loss of H3K4me3 occurs in another genome feature. To this end, we found decreased H3K4me3 signal in TEs, such as LINE1 and ERVL.

LINE1 constitutes about 17% of the human genome and is closely related to gene expression regulation and genomic stability [39]. Joanna et al. reported that LINE1 is critical for ZGA initiation by regulating global chromatin accessibility in mice [30]. In the present study, the H3K4me3 signal in LINE1 was decreased during ZGA in both mice and sheep. Consistently, the expression of LINE1 was decreased during ZGA in sheep. In mouse ESCs, depletion of Kdm5b leads to altered RNA Pol2 promoter occupancy and decreased RNA Pol2 initiation and elongation rates at active genes and at genes marked with broad H3K4me3 domains [22,24]. By re-analysis of previously published H3K4me3 ChIP data in these papers, we found increased H3K4me3 enrichment in LINE1 after Kdm5b knockdown. Specifically, RNA Pol2 occupancy in LINE1 was greatly improved. Considering that H3K4me3 regulates RNA polymerase II promoter-proximal pause–release [33,34], the enhanced RNA Pol2 occupancy might be mediated by H3K4me3.

## 4. Materials and Methods

### 4.1. In Vitro Maturation

In vitro maturation (IVM) was performed as previously described [6]. Briefly, sheep cumulus–oocyte complexes (COCs) with more than two layers of compact cumulus cells and a dense, homogeneous cytoplasm were obtained and cultured in groups of 20 in 75 μL droplets of IVM medium at 38.5 °C, with 5% CO_2_, 95% air, and saturated humidity for 20–22 h. MII-stage oocytes were collected for in vitro fertilization (IVF).

### 4.2. In Vitro Fertilization

Sheep IVF was performed as previously described [40]. Briefly, 20 mature oocytes were transferred to a 100 μL micro-drop of BO-IVF medium (#71004, IVF Bioscience, Falmouth, UK) with mineral oil covering the surface. Then, capacitated sperm were added to the micro-drops and cultured for 16 h at 38.5 °C, 5% CO_2_, and saturated humidity. The zygotes were transferred to the BO-IVC medium (#71005, IVF bioscience, Falmouth, UK) for further culture. The 2-, 4-, 8-, and 16-cell stage embryos, as well as the MII stage oocytes, morula, and blastocysts, were collected at defined points for CUT&Tag library preparation.

### 4.3. Embryonic Stem Cells Culture

Sheep ESCs were previously established in our lab (data in preparation). Briefly, sheep blastocysts were digested with pronase for 5 min to remove the zona pellucida. The inner cell mass was inoculated onto mitomycin C-treated mouse embryonic fibroblasts (MEFs) and cultured following the protocol for bovine-expanded potential stem cells [41]. Once cell colonies appeared, the cells were passed until the colonies stabilized and were digested with TrypLE when they reached 80% confluence, after which they were subcultured at a ratio of 1:10–12 onto a MEF feeder layer for further cultivation.

### 4.4. CUT&Tag Library Preparation and Sequencing

CUT&Tag library preparation was performed by NovoNGS CUT&Tag 4.0 High-Sensitivity Kit for Illumina (N259-YH01, Novoprotein, Beijing, China) following the manufacturer’s instructions. Briefly, embryos were removed from the zona pellucida by acidic Tyrode’s solution (T1788, Sigma-Aldrich, St. Louis, MO, USA) and washed with a wash buffer. The embryos were incubated with the anti-H3K4me3 (ab213224, 1:100 dilution; Abcam, Cambridge, UK) overnight at 4 °C, followed by incubation with ChiTag goat anti-rabbit IgG antibody (N269-01A, Novoprotein) for 1 h at RT. Following three washes with antibody buff, the embryos were treated with pAG-Transposome supplemented with 1% digitonin for 1 h at RT and transferred to a tagmentation solution for 1 h at 37 °C. DNA was extracted using tagment DNA extract beads and amplified with 15 cycles using i5 and i7 primers. The products were then sequenced on Illumina NovaSeq 6000 by Annoroad Gene Technology Company (Beijing, China).

### 4.5. CUT&Tag Analysis

The paired-end reads were aligned with the following parameters: --end-to-end --very-sensitive --no-mixed --no-discordant --phred33 -I 10 -X 1000 by Bowtie2 (v2.5.1). PCR duplicates were removed using picard (v2.27.5) with default parameters. For downstream analysis, the read counts were normalized by computing the number of reads per kilobase of bin per million reads mapped (RPKM) by deepTools (v3.5.1) using merged replicate bam files. To visualize the CUT&Tag signals in the UCSC genome browser, we generated the RPKM values on a 100 bp-window base using the bamCoverage function in deepTools. The metaplots were generated by computeMatrix and plotProfile functions in deepTools. Peaks of H3K4me3 were called using MACS2 with the parameters -B -f BAMPE --nomodel -nolambda -q 0.05 --keep-dup all --broad. Peaks annotation was performed using the ChIPseeker package (v1.38.0) of R software (v4.3.2). Gene Ontology (GO) analysis was performed by using the prcomp and clusterProfiler (v4.10.1) package of R software (v4.3.2). To identify the de novo motif for the H3K4me3 peaks, we employed the findMotifsGenome.pl function in HOMER (v4.11) with the default parameters.

### 4.6. ChIP-Seq Analysis

The published H3K4me3 ChIP-seq data of mouse embryos were downloaded from Gene Expression Omnibus (GEO) under accession number GSE73952 [7]. ESC H3K4me3 ChIP-seq data were downloaded from GEO under accession number GSE94739 [24]. H3K4me3 ChIP-seq data were analyzed with similar protocols in the CUT&Tag analysis.

### 4.7. RNA-Seq Analysis

The RNA-seq dataset of mouse pre-implantation embryos was downloaded from GEO under accession numbers GSE196520 [29] and GSE72784 [11]. RNA-seq data of Kdm5b knockdown ESC were downloaded from GEO under accession number GSE53090 [42]. The downloaded reads were trimmed to remove adaptors and low-quality bases using fastp (v0.23.4). Reads that passed quality control were mapped to reference genome mm39 using hisat2 (v2.1.0). All the mapped reads were then sorted and indexed using samtools (v1.9). Transcript quantification was performed through featureCounts (v2.0.1), and gene expression was normalized with fragments per kilobase of exon model per million mapped fragments (FPKM). Differentially expressed genes (DEGs) were identified using default parameters with the R package DESeq2 (v3.11), and genes with absolute log2 fold change >1 and statistical significance *p* value < 0.05 were deemed as DEGs.

### 4.8. Statistical Analysis

All statistical analyses were carried out in R. Quantifications are presented as mean values ± SEM (standard error of the mean). Statistical comparison was performed using the Wilcoxon test. Statistical significance, sample size, and the number of replicates were determined as indicated in the Figure legends. *p* values < 0.05 were regarded as significant (* *p* < 0.05; ** *p* < 0.01; *** *p* < 0.001).

### 4.9. Data Visualization

The R programming language, including the Bioconductor software packages (v3.18), was mainly used in the data visualization. The phylogenetic trees, Principal Component Analysis (PCA), heatmap, and boxplot were generated using the R packages ggtree (v3.17), ggrepel (v0.9.3), pheatmap (v1.0.12), and ggplot2 (v3.3.2), respectively. An integrative genomics viewer (IGV) browser was used to present the H3K4me3 signals at specific regions.

## 5. Conclusions

In summary, the broad H3K4me3 domains were removed while promoter H3K4me3 distribution was increased and correlated with gene upregulation during ZGA in sheep. We further reported the dynamic distribution of H3K4me3 at the TEs during early embryo development in both sheep and mice. Importantly, the RNA Pol2 distribution in TEs was greatly increased in Kdm5b knockout ESCs. Our data provide insights into the regulation of ZGA initiation and cell fate determination by H3K4me3.

## Data Availability

RNA-seq dataset was downloaded from GEO under accession numbers GSE196520, GSE72784, and GSE53090. Published H3K4me3 ChIP-seq data were downloaded from GEO under accession numbers GSE73952 and GSE94739.

## Supplementary Materials

The following supporting information can be downloaded at https://www.mdpi.com/article/10.3390/ijms252413545/s1.
